# Draft Genome Sequences of *Dickeya* sp. Isolates B16 (NIB Z 2098) and S1 (NIB Z 2099) Causing Soft Rot of *Phalaenopsis* Orchids

**DOI:** 10.1128/genomeA.00973-15

**Published:** 2015-09-10

**Authors:** Špela Alič, Tina Naglič, Pablo Llop, Nataša Toplak, Simon Koren, Maja Ravnikar, Tanja Dreo

**Affiliations:** a National Institute of Biology, Ljubljana, Slovenia; b Jožef Stefan International Postgraduate School, Ljubljana, Slovenia; c Centre of Excellence for Biosensors, Instrumentation and Process Control (COBIK), Laboratory for Bioanalytics (LBA), Ajdovščina, Slovenia; d Department of Evolutionary Genetics, Institute Cavanilles, University of Valencia, Paterna, Valencia, Spain; e OMEGA d.o.o., Ljubljana, Slovenia

## Abstract

The genus *Dickeya* contains bacteria causing soft rot of economically important crops and ornamental plants. Here, we report the draft genome sequences of two *Dickeya* sp. isolates from rotted leaves of *Phalaenopsis* orchids.

## GENOME ANNOUNCEMENT

Bacteria of the *Dickeya* genus of the family *Enterobacteriaceae* cause diseases on many plant species (1) and are currently divided into seven species: *D. dianthicola*, *D. dadantii*, *D. zeae*, *D. chrysanthemi*, *D. paradisiaca* (2, 3), *D. solani* (4), and *D. aquatica* (5). Several *Dickeya* sp. isolates form distinct clades and may represent novel species (1, 6, 7). Here, we report the draft sequences of two strains, *Dickeya* sp. B16 (NIB Z 2098) and S1 (NIB Z 2099), belonging to the undefined lineages UDL-3 and UDL-4 (1), which were identified as the primary cause of soft rot of *Phalaenopsis* orchids.

Both strains were purified by dilution plating. Genomic DNA was extracted using the UltraClean microbial DNA isolation kit (Mo Bio Laboratories, Inc., Carlsbad, CA) and concentrated to an approximate concentration of 500 ng/µl (GeneVac EZ-2plus; Genevac, Ipswich, United Kingdom), as determined with the Qubit double-stranded DNA (dsDNA) HR assay (Thermo Fisher Scientific, Life Technologies, Carlsbad, CA). Two single-read libraries were constructed with the Ion Xpress Plus fragment library kit (Life Technologies), according to the manufacturer’s protocol (8), with modifications: (i) 15 min DNA fragmentation time, (ii) six cycles in the library amplification, and (iii) library purification with 340 µl of Agencourt AMPure XP reagent (Beckman Coulter, Inc., Brea, CA). Fragments of approximately 150 to 200 bp were selected (E-Gel SizeSelect 2%; Life Technologies). Emulsion PCR was done with the Ion PGM template OT2 200 kit using the Ion OneTouch 2 system (both Life Technologies), according to the manufacturer’s protocol (9), with a final library concentration of 13 pM. The quality of the libraries was checked with the Caliper LabChip GX using the DNA high-sensitivity assay (PerkinElmer, Waltham, MA). Ion 316 Chip version 2 was used to sequence each library on the Ion Torrent Personal Genome Machine (PGM) sequencer with the Ion PGM sequencing 200 kit version 2 (all Life Technologies).

Sequences in FASTQ format were quality checked, and adapters were trimmed using CLC Genomics Workbench version 7.5 (Aarhus, Denmark), with the following modifications: (i) sequence quality score was set at 0.1, (ii) eight nucleotides were trimmed off every read at the 5′ terminal end, and (iii) reads of <15 nucleotides were discarded. Trimmed reads were independently assembled with CLC Genomics Workbench version 7.5 and the GS *de novo* assembler (454 Life Sciences, Roche Company, Branford, CT). Both assemblies were processed using standard program settings with minor modifications in the GS *de novo* assembler: (i) the all-contig threshold was set to 200 bp, and (ii) the long-contig threshold was changed to 1,000 bp. Assembled contigs were integrated using CISA from the Orione (Galaxy/CRS4) portal (http://orione.crs4.it/). Contigs with average coverage of <10×, contigs <1,000 bp, and all contaminates identified with BLASTn were discarded. The draft genomes comprise 61 and 59 contigs, with a total size of 4,885,657 bp and 4,892,969 bp and an *N*_50_ of 167,592 bp and 160,162 bp for strains B16 and S1, respectively.

### Nucleotide sequence accession numbers. 

The draft sequences of these two *Dickeya* strains are available in GenBank under the accession numbers JXBN00000000 (BioProject PRJNA268269) and JXBO00000000 (BioProject PRJNA268270) for B16 and S1, respectively. The versions described in this paper are the first versions, JXBN01000000 and JXBO01000000.
